# Historical prevalence of slavery predicts contemporary American gun ownership

**DOI:** 10.1093/pnasnexus/pgac117

**Published:** 2022-08-05

**Authors:** Nicholas Buttrick, Jessica Mazen

**Affiliations:** School of Public and International Affairs, Princeton University, Roberston Hall, Princeton, NJ 08540, USA; Department of Psychology, University of Wisconsin–Madison, Brogden Hall, Madison, WI, 53706, USA; Department of Psychology, University of Virginia, Gilmer Hall, Charlottesville, VA 22904, USA

**Keywords:** firearms, slavery, coping, safety, honor

## Abstract

American gun-owners, uniquely, view firearms as a means of keeping themselves safe from dangers both physical and psychological. We root this belief in the experience of White Southerners during Reconstruction—a moment when a massive upsurge in the availability of firearms co-occurred with a worldview threat from the emancipation and the political empowerment of Black Southerners. We show that the belief-complex formed in this historical moment shapes contemporary gun culture: The prevalence of slavery in a Southern county (measured in 1860) predicts the frequency of firearms in the present day. This relationship holds above and beyond a number of potential covariates, including contemporary crime rates, police spending, degree of racial segregation and inequality, socioeconomic conditions, and voting patterns in the 2016 Presidential election; and is partially mediated by the frequency of people in the county reporting that they generally do not feel safe. This Southern origin of gun culture may help to explain why we find that worries about safety do not predict county-level gun ownership outside of historically slave-owning counties, and why we find that social connection to historically slaveholding counties predicts county-level gun ownership, even outside of the South.

Significance Statement We suggest that the distinctly American belief that guns keep a person safe was partially formed in the backlash to Reconstruction after the American Civil War—a moment when a massive increase in the availability of firearms coincided with a destabilization of White politics in response to the emancipation and empowerment of Black Americans. We show that the historical prevalence of enslavement in a county predicts present-day frequency of firearms, and we show that the relationship between feeling unsafe and county-level firearms ownership is stronger in counties with a history of enslavement. Looking outside the South, we further show that social connection to historically slaveholding counties predicts firearm ownership.

## Introduction

Over 45% of all the civilian-owned weapons in the world are owned by the 5% of the world population that is American (1). Firearm-owners in America are distinct in how they think about their weapons: Over two-thirds report that they own a gun, at least in part, to keep themselves safe (2). Despite these beliefs, studies show that gun ownership doubles the likelihood that someone in the household will die in a violent homicide and triples the likelihood of a death by violent suicide (3), while offering little-to-no protection against assailants (4). These risks are understood by citizens of comparable nations, where people are more likely to think of firearms as dangerous than as safe (5, 6).

Why do so many Americans look to their firearms for safety? According to the Coping Model of Protective Gun Ownership, gun-owners use guns symbolically as an aid to manage psychological threats stemming from their belief that the world is a dangerous place from which society will not protect them (7, 8). American gun-owners are more likely than non-gun-owners to believe that the world is dangerous (9) and that institutions of order, such as government or police, are unable or unwilling to keep them safe (10). These beliefs trigger worries in gun owners concerning their fundamental needs, including their safety (11), their control and self-efficacy (12), and their place in society (13). Guns, in turn, become more salient to owners when core identities are threatened (14). Gun owners use their weapons to defend against all these meaning-threats (15), with owners more likely to believe that a gun keeps them safe (2), keeps them in control (16), and keeps them belonging to important social groups (17).

Where does this culturally unique belief that guns can be an effective coping mechanism come from? The belief that guns keep one safe was not widespread in the American antebellum era, where guns were more often viewed as tools (18). We argue that this changed during the Civil War. The end of the war and the demobilization of over half a million men, with their guns, left America as one of the most heavily armed societies in the world (19). With the destruction of the Southern economy after the war, these guns took on an important role. A contemporaneous estimate, for example, suggested that the value of the privately owned firearms in 1880s Alabama was significantly greater than the value of all mechanical tools and farm equipment in the state (20). This flood of weapons hit the South at an especially fraught moment. Historians and sociologists have argued that the destruction of the chattel slavery system in the South and the subsequent political and economic empowering of the previously enslaved Black population created an unstable system in which the political power of White elites was under existential threat, leading to a calculated backlash designed to maintain as much of that power as possible (21). As part of this so-called Redemption, White political leaders in the South argued that this New South, now led by Northern “carpetbaggers” and supported by the recently freed Black population, was a dangerous place uninterested in keeping White southerners safe [a contemporaneous estimate put the murder rate in the South as approximately 18 times greater than in New England, almost entirely driven by White-on-White or White-on-Black crime (22)], and therefore was in need of armed paramilitary organizations such as the Ku Klux Klan to maintain order where the government was unable to (23). White Southerners seemed to turn to their weapons as a means of dealing with this new world, especially when it came to political intimidation (24): Southern leaders explicitly anchored the protection of the Southern way of life in the private ownership of firearms, arguing that they protected (White) Southerners from an illegitimate government uninterested or unwilling to keep them safe. For a case study in this rhetorical strategy, we can look at the language used by the leaders of the failed 1874 plot to overthrow the New Orleans government. First, we have a speech by D.B. Penn, one of the leaders of the insurrection:

Through fraud and violence, the government of your choice has been overthrown and its power usurped…To these calamities, may be added a corrupt and vicious legislature,…a metropolitan police paid by the city, under the control of the usurper, quartered upon you to overawe and keep you in subjugation. Every public right has been denied you and, as if to goad you to desperation, private arms are seized… To such extremities are you driven that manhood revolts at further submission. (25)

His language was echoed by others in the movement, and we can see similar approaches to grounding Southern life in firearms ownership in petitions printed in contemporaneous newspapers (this one from J. Dickson Bruns, a leader in the Crescent City White League):

For nearly two years, you have been the silent but indignant sufferers of outrage after outrage heaped upon you by a usurping government. One by one, your dearest rights have been trampled upon, until, at last, in the supreme height of its insolence, this mockery of a republican government has dared even to deny you that right so solemnly guaranteed by the very Constitution of the United States, which in article two of the amendments declares that “the right of the people to keep and bear arms shall not be infringed.”…It now remains for us to ascertain whether this right any longer remains to us. We, therefore, call upon you…Declare That You Are Of Right, Ought To Be, And Mean To Be Free (26).

Firearms were not just used in symbolic defense of manhood in the post-war South. Northern observers at the time noted the importance of privately held arms in the White supremacist attempt to suppress Black political power and restore the antebellum status quo, reporting on institutions such as “rifle clubs,” which were aimed at “while avoiding actual bloodshed as much as possible, to so impress the blacks that they, or a number of them, will feel impelled to vote with the whites out of actual fear.” (27) Southern elites saw, in their guns, a means of protecting themselves and their interests from the social upheaval of Reconstruction, and they transmitted their beliefs to their Southern White brethren. We argue then that thanks to the sudden prevalence of firearms, which likely increased their salience, and the importance placed upon firearms by Southern leaders, White Southerners came to believe that a firearm was the sort of thing that kept one safe.

One might expect that this belief would have been especially concentrated in areas that had a particularly high degree of enslavement, as these would have been the areas that had the greatest degree of upheaval after Emancipation, and which worked hardest to retain White control over large now-freed Black populations (28). Social norms that are forged at transitional moments, where people are suddenly unsure about how to act and unsure about what is and will be appropriate in this new environment, can be especially potent and long-lasting (29, 30). Previous work has found that the cultural impact of slavery was powerful enough to be maintained intergenerationally, with contemporary residents of counties with higher rates of historical enslavement more likely to identify as conservative and more likely to report both implicit and explicit racial animus (31, 32). If the social pressures of Emancipation on White Southerners helped to create modern protective firearms culture in a similar manner, then we would expect those areas with a higher degree of enslavement before the Civil War to today show greater generalized worries about safety (even after controlling for objective measures of crime and policing), and as a result, have higher rates of firearms ownership.

Researchers have posited other roots for American gun culture. An additional explanation for a particularly Southern origin for gun culture places its beginnings in the Southern “culture of honor:” Psychologists have argued that the Southerners formed enduring norms that stressed the importance of maintaining a reputation for belligerence and responding swiftly and aggressively to threats—i.e. preserving one’s honor—as a means of protecting oneself in a world of weak centralized authority (33). A culture that places the responsibility for security in the arms of individual actors, and that lionizes the display of the potential for overwhelming retaliatory force would seem primed to seek out firearms as a means for protection, and researchers, in fact, have explicitly linked Southern patterns of protective firearms to the Southern culture of honor (34).

We set out to test whether Southern history of slavery helps to explain the creation of a worldview that motivates contemporary American gun ownership. We examine whether county-levels of historical enslavement predict contemporary weapons ownership in those counties, even after controlling for other sociopolitical residues that researchers have associated with American slavery, such as increased conservatism (31); increased ethnic fractionalization and increased crime (35); differential rates of education and income inequality along racial lines (36, 37); lower income (38); and decreased labor productivity (39); as well as testing whether the Southern history of slavery predicts contemporary gun ownership over and above prior explanations for Southern gun ownership such as the Southern culture of honor.

## Measuring firearms ownership

The United States does not formally track the number of weapons held by its population. To identify the county-level distribution of firearms in the United States, we use a tragic, but well-validated proxy measure: the percentage of suicides in the county that are committed with a firearm (40–43). Prior work validating this measure suggests that where gun ownership rates, as assessed by the General Social Survey or the International Crime Survey, are known, rates of suicide by firearm correlate with this objective measure *r* = 0.87 at the city level, *r* = 0.92 at the state level, and *r* = 0.95 cross-nationally (42), with the correlation at the county level not statistically distinguishable from an exact correlation measured with sampling error (41). Data on firearm suicides come from the CDC All-County Compressed Mortality Files, which record the death of every US resident. Our data covers the years 1999 to 2016, and are aggregated at the county level.

## Historical rates of enslavement and firearms ownership

**Fig. 1.**
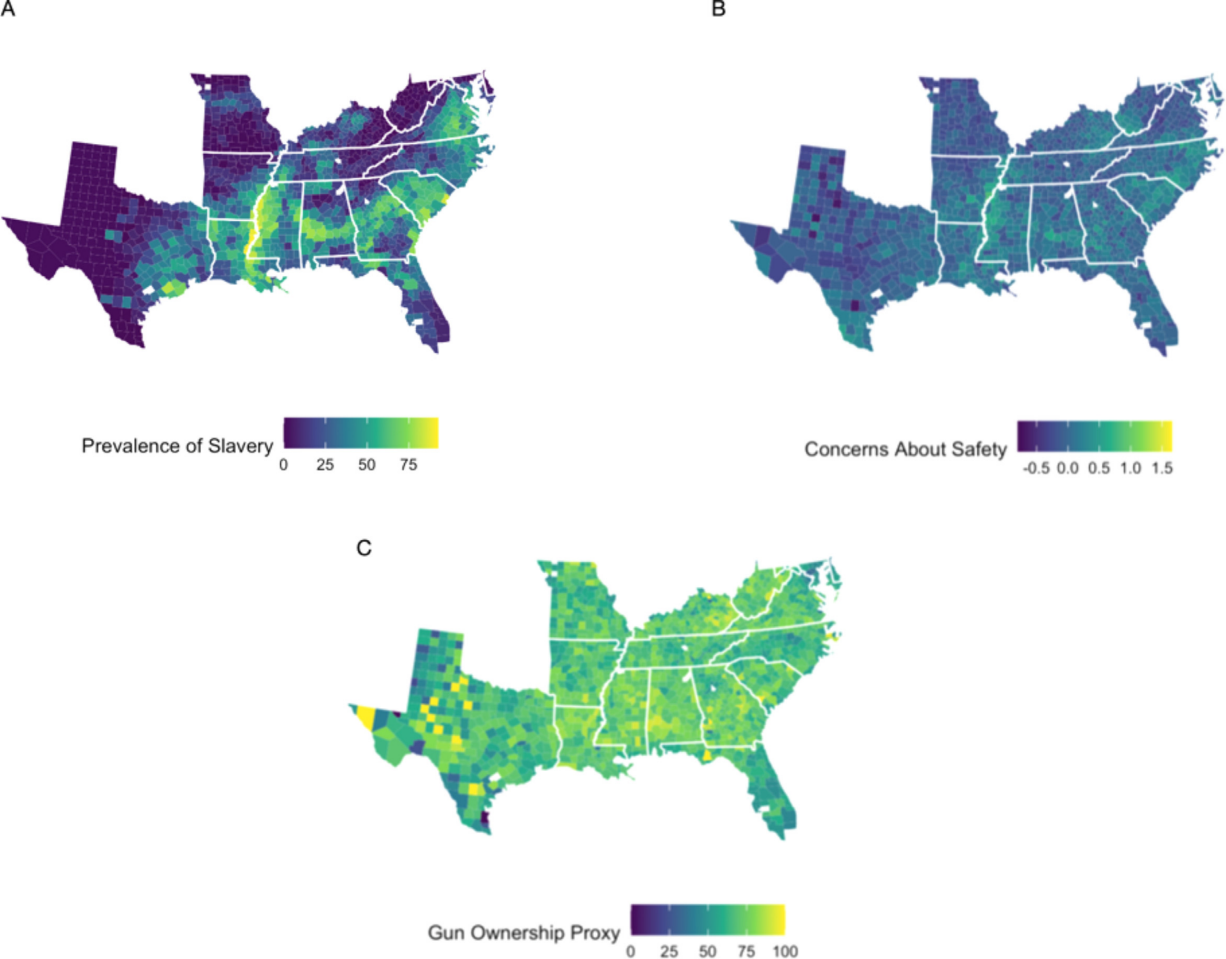
(A) Distribution of slavery in the 1860 census. (B) Contemporary self-reported worries about safety. (C) Gun ownership proxy (the ratio of suicides using a firearm to total suicides).

As our measure of the historical patterns of enslavement in the South, we use population data from the 1860 Census—the last census before the Civil War, which enumerated both enslaved and free Americans (37). “Southern” counties are defined as those where people were enslaved in 1860, including in states such as Kentucky, Maryland, and Delaware that were part of the Union (see Figure 1). As predicted, we find a relationship between the proportion of slaves in a county as a percentage of the total county population and the present-day ownership of firearms (1,509 counties): The higher the rates of historical enslavement in a county, the higher the rates of contemporary gun ownership, *b* = 0.034 [0.0030, 0.066], *se* = 0.016, *t*(1451) = 2.14, *P* = 0.032, *B* = 0.07 [0.01, 0.13]. Controlling for two classes of demographics: one set used by (31) to covary out sociodemographic differences between counties in 1860 (such as population, land quality, accessibility of rail and waterways, and the proportion of the county that was free Black) and the other based on contemporary differences between the counties (such as the poverty rate, degree of segregation, the effect of the contemporary Black population over and above historical patterns of enslavement, Black/White education disparities, income inequality, crime rate, spending on the police, votes for Donald Trump in the 2016 election, and the tightness of state gun laws; 1,123 counties in total), the intensity of enslavement in a county still positively predicts the present-day ownership of firearms, *b* = 0.13 [0.081, 0.19], *se* = 0.027, *t*(1014) = 4.86, *P* < 0.001, *B* = 0.30 [0.18, 0.42]. See Figure 1. See Table 1 for all standardized parameters for models that predict county-level patterns of firearms ownership from historical patterns of enslavement, including models without covariates, models with only the historical covariates, models with the contemporary covariates, and models with all covariates.

**Table 1.** Predicting county-level firearms ownership from historical patterns of slavery.

	Without controls	Without controls	1860 controls	Contemporary controls	All controls
Intensity of slavery	0.07* [0.01, 0.13]		0.22*** [0.12, 0.32]	0.23*** [0.15, 0.30]	0.30*** [0.18, 0.42]
Ruggedness of county (1860)		0.08** [0.03, 0.13]	0.06 [−0.00, 0.12]		0.09** [0.03, 0.15]
Squared county longitude			−0.02 [−0.19, 0.14]		−0.08 [−0.22, 0.06]
Squared county latitude			0.11 [−0.03, 0.26]		0.02 [−0.11, 0.15]
Log of county area			0.17*** [0.10, 0.24]		0.02 [−0.06, 0.09]
Inequality of land holdings (1860)			0.08* [0.01, 0.14]		−0.01 [−0.08, 0.05]
Proportion of farms under 50 acres (1860)			0.01 [−0.10, 0.12]		0.09 [−0.01, 0.19]
Log of county population (1860)			−0.31*** [−0.44, −0.18]		−0.09 [−0.23, 0.05]
Farm value per improved acre (1860)			−0.14*** [−0.20, −0.07]		−0.06* [−0.12, −0.00]
Log of total improved acres (1860)			0.15 [−0.01, 0.32]		0.17* [0.00, 0.33]
Proportion free Black (1860)			−0.12** [−0.19, −0.05]		−0.07 [−0.14, 0.00]
Rail access (1860)			−0.10*** [−0.15, −0.04]		0.02 [−0.04, 0.07]
Navigable waterway access (1860)			−0.09** [−0.15, −0.03]		−0.05 [−0.10, 0.00]
Proportion with at least a high-school education				0.04 [−0.04, 0.12]	0.04 [−0.04, 0.12]
Black/White high-school education ratio				−0.01 [−0.06, 0.04]	0.02 [−0.03, 0.07]
Residual proportion Black				0.12*** [0.05, 0.19]	0.22*** [0.13, 0.30]
Poverty rate				0.17** [0.06, 0.28]	0.18** [0.07, 0.30]
Racial segregation				−0.02 [−0.08, 0.04]	−0.04 [−0.10, 0.03]
Log of population density				−0.20*** [−0.28, −0.11]	−0.27*** [−0.37, −0.17]
Household income per capita				0.06 [−0.03, 0.15]	0.06 [−0.04, 0.15]
Income inequality				−0.01 [−0.08, 0.05]	0.01 [−0.06, 0.07]
Crime rate				0.01 [−0.07, 0.10]	0.03 [−0.06, 0.11]
Violent crime rate				0.02 [−0.06, 0.10]	−0.01 [−0.09, 0.08]
Labor force participation				−0.03 [−0.12, 0.05]	0.04 [−0.05, 0.13]
Local government expenditures per capita				−0.06* [−0.11, −0.01]	−0.01 [−0.06, 0.05]
Unemployment rate				0.06 [−0.01, 0.12]	0.07* [0.00, 0.14]
Social capital index				−0.04 [−0.10, 0.02]	−0.03 [−0.09, 0.03]
Police spending/total wage expenditures				0.00 [−0.06, 0.06]	0.02 [−0.04, 0.09]
Proportion Trump vote, 2016				0.36*** [0.27, 0.44]	0.41*** [0.31, 0.51]
Strictness of state gun laws				−0.11* [−0.20, −0.02]	−0.10 [−0.20, −0.00]
Counties	1,509	1,408	1,285	1,268	1,123

Note: All estimates are standardized betas. 95% confidence intervals are in brackets. *P < 0.05; **P < 0.01; ***P < 0.001.

### Alternate explanation: the culture of honor

What about the argument that Southern gun ownership is driven by a culture of honor? To measure the presence of an honor culture in a county, we adopt an ecologically based operationalization used in prior literature: the ruggedness of that county. More rugged counties, it is theorized, were more conducive to herding than farming, and as livestock were more susceptible to theft than other forms of agriculture, an economy based in herding required the creation of individualized reputation-based approaches to protecting property that are at the core of the culture of honor (see 44 and 45 for a similar operationalization). We find that the power of ruggedness to predict county-level gun ownership is present, but weaker than for the intensity of slavery. When we predict gun ownership by county-level ruggedness, we find that it positively predicts present-day gun ownership (in 1,408 counties), *b* = 0.018 [0.0057, 0.29], *se* = 0.0060, *t*(1405) = 2.92, *P* = 0.004, *B* = 0.08 [0.03, 0.13]. However, when we simultaneously predict gun ownership by both county-level ruggedness and the presence of slavery in a county (including all county-level covariates, 1,123 counties in all), we find that the effect of ruggedness, *b* = 0.018 [0.0072, 0.031], *se* = 0.0061, *t*(1065) = 3.10, *P* = 0.002, *B* = 0.09 [0.03, 0.15], is smaller than the effect of slavery, *B* = 0.30 [0.18, 0.42] (see Table 1, column 5; see the online supplement for additional analyses of the relationship between ruggedness and historical intensity of enslavement).

### Additional robustness checks: white gun owners

As a robustness check, we restricted our gun-ownership proxy to just suicides committed by non-Hispanic Whites. The all-demographic gun ownership proxy used above and the White-only proxy are highly correlated at the county level, *r*(3212) = 0.915 [0.91, 0.92], *P* < 0.001, and we find that our relationship in Southern counties between the intensity of historical enslavement in a county and the present-day ownership of firearms by Whites is largely unchanged: without covariates (1,509 counties), *b* = 0.052 [0.020, 0.085], *se* = 0.017, *t*(1422) = 3.14, *P* = 0.002, *B* = 0.10 [0.04, 0.16]; with all covariates (1,123 counties), *b* = 0.068 [0.014, 0.12], *se* = 0.028, *t*(991.9) = 2.38, *P* = 0.017, *B* = 0.15 [0.03, 0.27]. See Tables S1 and S2 for the full regression tables from the White-only models, as well as models restricted to counties with greater than 25,000 people (following the firearm ownership identification strategy of 42).

## The mediating role of feeling “unsafe”

Coping models of protective gun ownership suggest that people own firearms as a means of dealing with perceived threats that make them feel unsafe in their environment (7, 8). We examined, therefore, whether areas in the South with a history of more intense enslavement have present-day residents who feel more unsafe, and whether this feeling of safety mediates the relationship between historical patterns of enslavement and present-day gun ownership.

To measure current-day feelings, we used data from the Gallup Daily Tracking Poll, which uses random-digit dialing to survey roughly 1,000 Americans daily about their psychological state and well-being. Data comes from the years 2008 to 2017, aggregated at the county level, and contains over 3.6 million respondents. In the analyses below, we restrict our sample to those counties with at least 100 responses in our dataset, though we report models with all counties in the online supplement (see Tables S3 to S6).

As predicted, we find, controlling for both our 1860 and contemporary covariates (1,044 counties in total), that counties in the South with a history of more intense enslavement are less likely to feel safe in the present day, *b* = −0.0044 [−0.0051, −0.0035], *se* = 0.0042, *t*(956.3) = −10.27, *P* < 0.001, *B* = −0.49 [−0.59, −0.40], and that lacking this sense of safety predicts gun ownership, *b* = −7.49 [−11.17, −3.58], *se* = 1.96, *t*(1012) = −3.83, *P* < 0.001, *B* = −0.15 [−0.23, −0.07], with safety mediating the relationship between counties with a higher proportion of slaves and present-day gun ownership, average mediation = 0.032 [.015, 0.051], *P* < 0.001; direct effect, *b* = 0.11 [.057, 0.16], *P* < 0.001, total effect, *b* = 0.14 [.090, 0.19], *P* < 0.001. Using those same controls and counties, we find that the ruggedness of a county in 1860 does not predict contemporary feelings of safety in those counties (*P* = 0.83), and that feelings of safety therefore do not mediate a relationship between the ruggedness of a county in 1860 and the present-day distribution of firearms, *P* = 0.80. See Table S3 for tests of two alternate mediators: contemporary daily feelings of anger, and sense of self-respect (operationalized as feeling like one is able to use one’s strengths daily). We find no evidence for either mediator.

### Geographic specificity

**Fig. 2.**
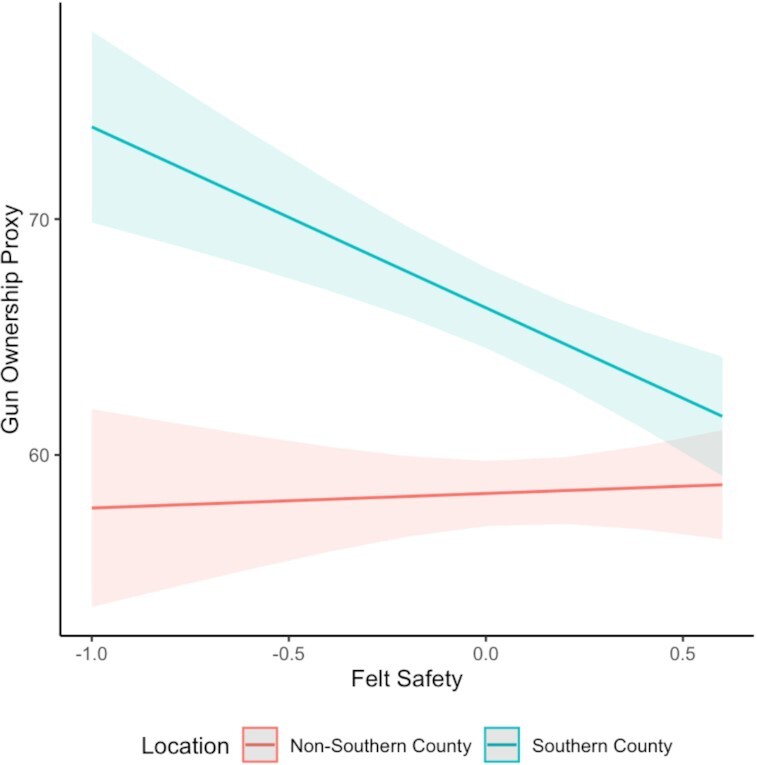
County-level feelings of safety predicting county-level gun ownership in Southern and non-Southern counties.

Importantly, we find that the relationship between safety-threat and gun-ownership behavior is largely restricted to Southern counties. Comparing Southern counties with non-Southern counties and controlling for our contemporary set of covariates (2,308 counties), we find that in the South, counties that collectively report feeling less safe have greater rates of gun ownership, marginal trend: *b* = −7.67 [−11.15, −4.19], *B* = −0.13 [−0.19, −0.071], while for non-Southern counties, there is no relationship between county-level feelings of safety and gun ownership: *b* = 0.62 [−3.05, 4.28], *B* = 0.010 [−0.052, 0.073]; interaction: *b* = −8.29 [−12.38, −4.13], *se* = 2.11, *t*(2283) = −3.93, *P* < 0.001, *B* = −0.14 [−0.21, −0.07] (see Figure 2). Restricting our gun-ownership proxy to Whites does not change our conclusions. See Table S7 and Figure S1 for the full regression tables, as well as models without controls, models using all counties, models restricted to White gun owners, and models restricted to counties with more than 25,000 people.

## Social diffusion of firearm/safety beliefs

We next sought to understand how gun culture diffused throughout the broader United States, in order to explain the fact that contemporary gun ownership is not strictly concentrated in Southern counties. We argue for social transmission of belief as one possible vector for the spread of firearms throughout the country. If patterns of migration were to explain the diffusion of gun culture out of the South throughout the United States, we would expect that counties throughout the country with deeper social ties to areas of historically intense enslavement would be more likely to own firearms. To measure the degree of social-connectedness, we used data from the Facebook Social Connectedness Index, which calculates the relative probability that any two people in two different counties are friends on Facebook, and which therefore allows us to map the density of social ties between any two counties in the United States (46). We constructed an enslavement-connection index for each county by multiplying the strength of social connection to each other county by the intensity of enslavement in the connected county, and then summing up all the products. We also constructed a parallel index, measuring the strength of social ties to counties that have more firearms in the present day, as a way of testing whether patterns of gun ownership are better understood as arising from contemporary social transmission (as opposed to our historical explanation). All indices were log-transformed to address skewness.

We find that the degree of social connectedness with counties that had high rates of historical enslavement predicts gun ownership above and beyond the county’s degree of social connectedness with other counties that have high rates of contemporary gun ownership (using 3,213 counties), *b* = 1.03 [0.33, 1.89], *se* = 0.39, *t*(763.02) = 2.83, *P* = 0.005, *B* = 0.11 [0.03, 0.19]. Additionally controlling for our set of contemporary covariates (using 2,609 counties), we still find that connectedness to counties with high rates of historical enslavement predicts contemporary gun ownership above and beyond connection to other counties with high levels of contemporary gun ownership, *b* = 2.31 [1.51, 3.16], *se* = 0.42, *t*(310.7) = 5.55, *P* < 0.001, *B* = 0.26 [0.17, 0.36]. When we restrict our analysis to counties with no history of enslavement (those mainly in the North and West), we nevertheless find that the more connected these counties are with those counties that had higher rates of historical enslavement, the higher the rates of county-level gun ownership (using 1,341 counties, and all contemporary controls) *b* = 2.46 [0.48, 4.52], *se* = 1.042, *t*(692.2) = 2.36, *P* = 0.019, *B* = 0.08 [0.01, 0.15]. See Table 2 for the standardized coefficients of all models looking at social connectedness to patterns of enslavement predicting county-level patterns of firearm ownership, both with and without contemporary controls. Results are directionally consistent when restricting to the White-only gun ownership proxy. See Tables S8 and S9 for models restricted to the White-only proxy, and counties greater than 25,000 people.

**Table 2.** Predicting county-level firearm ownership from social-connectedness indices.

	Without controls	With controls	Without controls	With controls	Non-Southern, without controls	Non-Southern, with controls
Slavery connectedness index	0.11** [0.03, 0.19]	0.26*** [0.17, 0.36]	0.11** [0.03, 0.19]	0.26*** [0.17, 0.35]	0.08** [0.03, 0.14]	0.08* [0.01, 0.15]
Gun connectedness index	0.46*** [0.42, 0.51]	−0.03 [−0.11, 0.05]	0.46*** [0.42, 0.51]	−0.03 [−0.11, 0.04]	0.59*** [0.52, 0.66]	0.02 [−0.11, 0.15]
Frontier connectedness index			0.00 [−0.04, 0.04]	0.02 [−0.02, 0.06]	0.01 [−0.05, 0.06]	−0.01 [−0.07, 0.04]
Proportion with at least a high-school education		0.05* [0.00, 0.10]		0.05 [−0.00, 0.10]		0.04 [−0.02, 0.10]
Black/White high-school education ratio		−0.01 [−0.04, 0.02]		−0.01 [−0.04, 0.02]		−0.01 [−0.05, 0.03]
Proportion Black		0.11** [0.04, 0.18]		0.10** [0.03, 0.17]		0.06 [−0.01, 0.13]
Poverty rate		−0.04 [−0.11, 0.02]		−0.05 [−0.12, 0.02]		−0.19*** [−0.28, −0.10]
Racial segregation		−0.10*** [−0.13, −0.06]		−0.09*** [−0.13, −0.06]		−0.13*** [−0.19, −0.07]
Log of population density		−0.27*** [−0.33, −0.21]		−0.27*** [−0.34, −0.21]		−0.40*** [−0.50, −0.30]
Household income per capita		−0.03 [−0.08, 0.03]		−0.03 [−0.08, 0.03]		−0.09* [−0.17, −0.02]
Income inequality		0.05* [0.00, 0.09]		0.05* [0.00, 0.09]		0.06 [−0.00, 0.11]
Crime rate		0.01 [−0.04, 0.06]		0.01 [−0.04, 0.06]		0.01 [−0.06, 0.08]
Violent crime rate		−0.01 [−0.06, 0.04]		−0.01 [−0.06, 0.04]		−0.06 [−0.13, 0.02]
Labor force participation		−0.03 [−0.09, 0.02]		−0.03 [−0.09, 0.02]		−0.04 [−0.10, 0.03]
Local government expenditures per capita		−0.02 [−0.05, 0.02]		−0.02 [−0.05, 0.02]		−0.01 [−0.07, 0.04]
Unemployment rate		0.10*** [0.06, 0.15]		0.10*** [0.06, 0.15]		0.14*** [0.08, 0.21]
Social capital index		−0.01 [−0.07, 0.04]		−0.01 [−0.06, 0.04]		−0.00 [−0.07, 0.07]
Police spending/total wage expenditures		0.01 [−0.03, 0.04]		0.01 [−0.02, 0.04]		0.01 [−0.03, 0.06]
Proportion Trump vote, 2016		0.25*** [0.20, 0.30]		0.25*** [0.20, 0.30]		0.18*** [0.10, 0.26]
Strictness of state gun laws		−0.15*** [−0.22, −0.08]		−0.15*** [−0.22, −0.08]		−0.12* [−0.22, −0.01]
Counties	3,213	2,609	3,213	2,609	1,704	1,341

Note: All estimates are standardized betas. 95% confidence intervals are in brackets. *P < 0.05; **P < 0.01; ***P < 0.001.

**Fig. 3.**
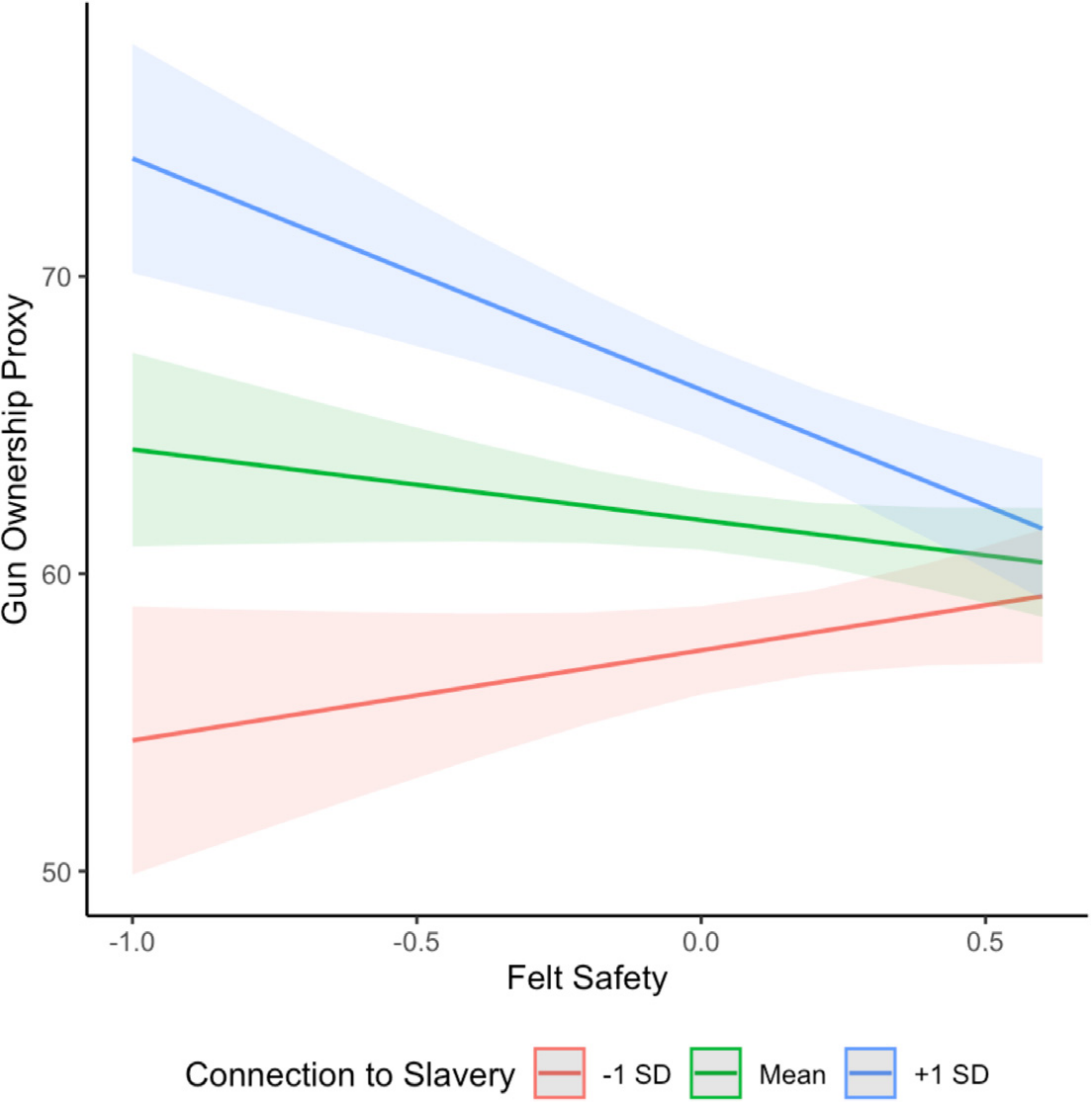
County-level feelings of safety predicting county-level gun ownership in counties with a high degree of connection to counties with a greater history of enslavement (+1 SD), a moderate degree of connection (Mean), and a lower degree of connection (−1 SD).

Finally, we investigated whether the degree to which feelings of safety predict gun ownership is moderated by how connected people in that county are to counties with high rates of historical enslavement. We find that it is: The more connected a county is to a county that had high rates of historical enslavement (controlling for patterns of social connectedness to counties with high rates of contemporary gun ownership, and our set of contemporary covariates, and restricting the sample to counties with at least 100 respondents to the Gallup Daily Tracking Poll; 2,308 counties in total), the more likely that low ratings of felt safety predicted high levels of gun ownership: at 1 SD above the mean, marginal trend: *b* = −7.71 [−11.04, −4.38], *B* = −0.13 [−0.19, −0.075], while counties with less of a connection to counties with high rates of historical enslavement did not show any relationship between felt safety and gun ownership: at 1 SD below the mean, marginal trend: *b* = 2.94 [−0.82, 6.71], *B* = 0.051 [−0.013, 0.12], interaction: *b* = −3.68 [−5.08, −2.28], *se* = 0.72, *t*(2282) = −5.14, *P* < 0.001, *B* = −0.09 [−0.13, −0.06]. Conclusions are unchanged when using the White-only gun-ownership proxy. See Figure 3, and see Table S10 and Figure S2 for the full regression tables, models using the White-only gun-ownership proxy, models using all counties, models without controls, and models restricted to counties greater than 25,000 people.

As an additional robustness check, following the hypotheses of e.g. (47) and (48) that American gun culture can be traced not to the South but rather to its connection with the Frontier, we additionally tested the relationship between social connection to the Frontier and contemporary gun ownership. We found no evidence for an association between the two. See supplemental materials for more information.

## Limitations and conclusions

There are clear limitations to the current work, largely due to the reluctance of the US government to track rates of firearms ownership. Because the use of a proxy is required to estimate firearm ownership rates, we have a limited ability to disambiguate weapons ownership for particular demographics that make up smaller minorities within a county. There are, for example, likely not enough Black gun suicides in most counties to allow us a clearer picture of Black gun ownership throughout the country, especially in nonurban counties (49). This general reluctance to ask about weapons ownership in national surveys additionally means that we are only able to track beliefs at the county-level, not within individual respondents (though see e.g. 31 and 32 for evidence of the validity of county-level aggregation of individual psychological variables). While this use of a proxy adds unavoidable noise to our findings, noise that is compounded by an attempt to assess relationships over a century-and-a-half of history, we nevertheless note that our findings are robust to a number of alternate specifications and analytic choices.

We also do not wish to suggest that historical patterns of enslavement are the only determinant of current-day patterns of firearms ownership. Following prior work, we do, for example, find that the ecological roots of honor–culture formation independently predict patterns of contemporary firearms ownership, and it may be the case that other aspects of American history and demography have influenced the creation of contemporary American gun culture, including, for example, honor cultures that may have formed in the wake of Reconstruction. The percentage of contemporary patterns of firearms ownership that is uniquely explainable by historical patterns of enslavement in our maximal models is fairly small (*epsilon^2^ =* 0.02 [0.01, 0.04]), but we find that it is, for example, not significantly different in magnitude than the percentage explained by how much of the county voted for Donald Trump in the 2016 election (*epsilon^2^* = 0.06 [0.03, 0.09]).

If the use of weapons as a coping mechanism has some of its roots in an exclusionary, anti-Black regime, it may be no surprise that American gun rights are often coded as something exclusively for and about White Americans, both explicitly and implicitly (50, 51); that gun laws are often selectively used to prevent Black Americans, specifically, from owning guns (52); that racial resentment predicts opposition to gun control in White Americans (50); that leadership of the Gun Owners of America, a major gun-rights organization, grounded their movement in an explicitly White-supremacist ideology (53); that racially resentful White Americans become *more* supportive of gun control when informed that Black Americans are arming themselves faster than Whites (51); that in areas with more non-White people, study participants have a lower threshold to shoot Black targets in a shooter-bias paradigm (54); and that racism is associated with an increased likelihood of gun ownership among Whites (55).

Contemporary American gun politics are an international outlier. American gun laws are far more lax than other developed nations (56), and opposition to the laws that do exist is often grounded in the belief that guns provide safety to their owners (57). We argue that this belief in the protective power of weapons was crystallized during the fight of White Southerners to reclaim their privileges after the collapse of the slaveholding society precipitated by the loss of the Civil War, which may explain why the link between feeling unsafe and owning a gun is so much stronger in the South than in the rest of the country, and why social connection to historically slaveholding counties predicts contemporary firearms ownership. The American psychology around protective weapons ownership, in other words, is not an accident—we argue that it is a belief system grounded in and formed by a response to one of the signal events of American history.

## Materials and methods

### Data sources

Data for the historical prevalence of slavery in Southern counties come from the 1860 US Census, with borders updated by (37). Data for enslavement in the state of Missouri, along with the 1860 covariates come from (31), and can be found at https://dataverse.harvard.edu/file.xhtml?persistentId=doi:10.7910/DVN/CAEEG7/IAHLGX&version = 1.0. In the Southern counties, where the (37) and (31) slavery datasets overlap, the correlation between the estimates generated by their two slightly differing approaches to updating county borders in order to match contemporary divisions is quite high: *r*(1276) = 0.988 [0.986, 0.989]. Data on county-level firearm suicides come from the CDC All-County Mortality Files (see https://www.cdc.gov/nchs/data_access/cmf.htm for access). Data from the Gallup Daily Tracking Poll can be accessed through Gallup Analytics. Educational information comes from the 2016 5-Year American Community Survey. 2016 presidential voting patterns come from the Atlas of US Presidential Elections (https://uselectionatlas.org/). Data on police spending come from the 2017 US Census State and Local Government Finance Datasets (https://www.census.gov/data/datasets/2017/econ/local/public-use-datasets.html). Data on the social connectedness of counties come from the Facebook Connectome (https://data.humdata.org/dataset/social-connectedness-index, see (46) for additional details. Data on county-level exposure to the Frontier come from (58). Gun law data come from the 2013 state ratings of (59). All other contemporary covariates come from (60); the codebook can be found at https://opportunityinsights.org/wp-content/uploads/2018/04/online_table4-2.pdf). A precise description of all variables, and their sources can be found in Table S12. Due to data-use agreements with the CDC and the Gallup Organization, we are unable to share our raw data files.

### Analytic approach

All analyses were conducted in R. We constructed multilevel regression models, nesting counties within states, using the *lme4* and *lmerTest* packages. Descriptive statistics for all variables can be found in Table S11, and intercorrelations between all variables are presented in Figure S3. Mediation models similarly nested counties within states, using the *mediation* package. Measures based on the rate of suicide by firearm, and on the Gallup Daily Tracking Poll were created by aggregating data within county, collapsing across years. Social-connectedness indices were constructed for each county by first taking the relative probability that any person in a target county would be friends on Facebook with a person in the connected county, and then multiplying that probability with historical levels of enslavement in the connected county. To get a measure of total social exposure to historical patterns of enslavement in a target county, we summed up these products across every county that a target county was socially connected to, and then log-scored the sum. We constructed matching indices, using the same approach, for connection to patterns of contemporary gun ownership and for connection to the Frontier. For models that contain both county-level intensity of slavery and the contemporary county-level proportion of Black residents, we enter in the residual of contemporary Black population not explained by historical patterns of enslavement, as, due to trends in population migration, the two variables correlate very strongly (*r* = 0.77 [0.75, 0.79]). Conceptually, this assigns the high degree of shared variance between the two indices to historical patterns of slavery, which we think is reasonable due to temporal priority, and therefore the coefficient assigned to the residual contemporary Black population can be interpreted as the effect of the contemporary Black population over and above historical patterns of enslavement. See the online supplement for additional robustness checks for this interpretation. See https://osf.io/sgc9a for all analysis scripts.

## Supplementary Material

pgac117_Supplemental_Files

## Data Availability

Materials for this article can be found at https://osf.io/uxt3q/and data sources are listed in Table S12. Due to data-use agreements with the CDC and the Gallup Organization, we are unable to directly share raw data.
