# Supplementary material for: Historical prevalence of slavery predicts contemporary American gun ownership
Source: PNAS Nexus. 2022 Aug 5;1(3):pgac117. doi: 10.1093/pnasnexus/pgac117 (PMC9896914; doi:10.1093/pnasnexus/pgac117)
Supplement: pgac117_Supplemental_Files [file pgac117_supplemental_files.zip › PNASNEXUS-PNASNEXUS-2021-00239-s01.docx]

Supplementary Materials for:

Historical prevalence of slavery predicts contemporary American gun ownership

Nicholas Buttrick, Jessica Mazen.

Correspondence to: [buttrick@princeton.edu](mailto:buttrick@princeton.edu)

**This PDF file includes:**

Supplementary Text

Figs. S1 to S3

Tables S1 to S12

**Supplementary Text**

Testing the Predictive Power of the Frontier

One alternate model of American gun culture places its locus in exposure to the norms of the Frontier. According to this narrative, firearms were needed to protect oneself in the Wild West and this independent frontier spirit helped to popularize firearms throughout the nation, setting the template through which future generations understood the utility of a gun (e.g. *47, 48*). We therefore additionally test whether present-day social connectedness to counties that spent more time as part of the American frontier instead explains the pattern of contemporary gun ownership. To measure the “frontierness” of a county from 1790-1890 (1890 being the date of the official ‘closing of the frontier,’ per the U.S. Census), we use a measure from *(58)* that tracks the number of years that a county was both geographically close to the frontier (the line dividing counties with more than two people per square mile from those less densely populated) and was itself populated with fewer than six people per square mile. We then constructed a social connectedness index to these counties matching the social-connection indices in the main text.

We find that social connection to a frontier county does not help to explain current trends in firearm ownership - in a model predicting gun ownership from social connectedness to counties with high rates of historical enslavement, counties with high rates of historical frontier exposure, and counties with high rates of contemporary gun ownership (using 3,213 counties in total), we find that rates of historical enslavement predicts present day gun ownership, *b* = 1.39 [0.41, 1.89], *se* = 0.40, *t*(794.7) = 2.76, *p* = .006, *B* = 0.11 [0.03, 0.19]; whereas rates of historical frontier exposure does not, *b* = 0.068 [-0.58, 0.72], *se* = 0.33, *t*(3163.4) = 0.21, *p* = 0.84, *B* = 0.00 [-0.04, 0.04]. Additionally controlling for our set of contemporary covariates (2,609 counties) does not change this pattern of results, as connectedness with counties with high rates of historical enslavement still predicts contemporary gun ownership, *b* = 2.27 [1.47, 3.12], *se* = 0.42, *t*(3123) = 5.44, *p* < .001, *B* = 0.26 [0.17, 0.35]; while rates of connectedness with counties with high rates of frontier exposure does not, *b* = 0.32 [-0.24, 0.89], *se* = 0.29, *t*(1614) = 1.12, *p* = .26, *B* = 0.02 [-0.02, 0.06]. See Table 2 for the full output. Conclusions from models restricted to the White-only proxy do not differ, see Tables S8.

This lack of evidence may not come as a surprise to those historians who have argued that “frontier gun culture” was largely a revisionist account, motivated by the later advertising agencies of the gun manufacturers *(19)* or by a political and intellectual culture searching the past for a unifying national mythology *(61)*.

The Relationship Between Cultures of Honor and Historical Patterns of Enslavement

How are cultures of honor related to historical patterns of enslavement? Are they part of the same gun-ownership phenomenon, or are they contributing in different ways? In the main text, we find that both the ruggedness of a county (as a proxy for those counties likely to develop honor cultures) and the historical intensity of enslavement both predict contemporary gun ownership behavior independent of each other. We ran additional analyses to better understand how the two constructs relate to each other and how they jointly predict gun ownership.

Firstly, we find that the relative magnitudes of the relationship between historical patterns of enslavement and contemporary gun ownership is still stronger than the relationship between ruggedness and contemporary gun ownership when the models are based on the same underlying sample of counties: in the counties where all data is available for both measures, we find that the predictive effect of slavery on gun ownership, without including ruggedness in the model is *B* = 0.22 [0.11, 0.33]; the predictive effect of ruggedness on gun ownership without including slavery in the model is *B* = 0.03 [-0.02, 0.09]; and, as mentioned in the main text, in a model containing both predictors, the strength of relationship of slavery is *B* = 0.30 [0.18, 0.42] while the strength of relationship of ruggedness is *B* = 0.09 [0.03, 0.15].

In looking at the relationship between the two constructs, perhaps unsurprisingly, given the ecological demands of plantation agriculture *(62)*, we find that counties with historically higher patterns of enslavement are less likely to be rugged, *r*(1405) = -.38 [-.42, -.33], p < .001. When looking at the ways in which the ecological conditions conducive for cultures of honor interact with historical patterns of enslavement, we find that relationship between historical patterns of enslavement and contemporary gun ownership is somewhat stronger in less rugged areas (-1 SD ruggedness, *B* = 0.17 [0.082, 0.25]) than it is in areas that were more rugged (+1 SD ruggedness, *B* = 0.0081 [-0.12, 0.14]; interaction *b* = -0.00082 [-0.0016, -0.000016], *se* = 0.00041, *t*(1397) = -1.96 *p* = .046). That enslavement is more strongly connected to gun ownership in areas likely to have weaker honor cultures suggests to us that historical patterns of enslavement and the culture of honor may be relatively distinct.

We are loath to make the argument that cultures of honor are completely separate from historical patterns of enslavement, given the weakness of the interaction and the cross-sectionality of this part of our data; since both our ruggedness measure and our enslavement measure come from 1860, we have no data on the temporal relationship between cultures of honor and enslavement, and so cannot say how one did or did not foster the other. We would be glad to see more in-depth ethnographic research on the relationship between cultures of enslavement and cultures of honor, especially analyses that can quantify the temporal development of honor cultures in the post-Reconstruction South *(e.g., 33)*.

Testing Between Different Explanations for the Role of Race in Our Models.

In the analyses of Southern states in main text of the paper, for models that contain both county-level intensity of slavery and the contemporary county-level proportion of Black residents, we enter in the residual of contemporary Black population not explained by historical patterns of enslavement, as, due to trends in population migration, the two variables correlate very strongly (*r* = .77 [.75, .79]). Conceptually, this assigns the high degree of shared variance between the two indices to historical patterns of slavery, which we think is reasonable due to temporal priority, and therefore the coefficient assigned to the residual contemporary Black population can be interpreted as the effect of the contemporary Black population over and above historical patterns of enslavement. This leaves open the possibility, however, that our models are simply picking up on a fully contemporary relationship – that this shared variance should instead be assigned to the contemporary Black population, and that therefore our models are showing that contemporary Americans gun ownership strategies are simply sensitive to the contemporary racial makeup of their county. To try to disambiguate between these two interpretations of the models, we ran three additional analyses to investigate whether patterns of gun ownership are related not just to the contemporary Black population in a county, but, as we have hypothesized, to historical patterns of enslavement.

Firstly, we tested whether the South is different from the rest of the country when it comes to the relationship between gun ownership and the contemporary Black population. If contemporary gun ownership is simply a function of the presence of Black Americans, then this relationship should not differ across these two regions, but if gun ownership is instead influenced by historical patterns of enslavement, then there should be a stronger relationship in the South.

We find a significant interaction in the relationship between our gun ownership proxy and the current-day Black population in counties in the South versus counties in the rest of the country. In the South, a greater number of Black residents in a county predicts increased gun ownership in that county (*B* = 0.058 [0.012, 0.11]), while outside of the South, a greater number of Black residents predicts decreased gun ownership in that county (*B* = -0.66 [-0.82, -0.49]; interaction *b* = 66.11 [52.06, 80.27], *se* = 7.20, *t*(3007.55) = 9.19, *p* < .001). The increased presence of Black Americans in a county does seem to predict different patterns of gun ownership in the South than everywhere else in the United States.

Within the South, moreover, we find that the relationship between the Black population and the ownership of firearms is stronger in counties with higher rates of enslavement (+1 SD, *B* = 0.062 [-0.018, 0.14]) than in counties with lower rates of enslavement (-1 SD, *B* = -0.11 [-0.23, 0.016]; interaction *b* = 0.25 [0.10, 0.40], *se* = 0.077, *t*(1502.92) = 3.25, *p* = .001. Southerners living in historically slaveholding areas seem to react differently to the presence of Black Americans than do Southerners living in areas with less historical enslavement.

Finally, looking just outside the South, in counties with less connection to historically-slaveholding counties, a higher number of Black residents predicts decreased firearm ownership (-1 SD social connection to the South *B* = -0.47 [-0.56, -0.38]), a relationship which is weaker in those non-Southern counties with a stronger connection to historically-slaveholding counties (+1 SD social connection to the South *B* = -0.25 [-0.30, -0.20]; interaction *b* = 92.98 [65.49, 120.82], *se* = 14.08, *t*(1616.85) = 6.60, *p* < .001). Counties that are more socially connected to slaveholding react differently to the presence of Black Americans than do counties with less connection to historical enslavement.

Given this picture, then, that race seems to predict firearms ownership differently between the historically slave-owning South and the non-slave-owning rest of the nation, that race predicts firearms ownership differently within the South as a function of the historical presence of slavery, and that historical connection to slavery seems to matter when assessing the relationship between race and firearms ownership in the non-Southern United States, we think that the balance of evidence is in favor of our historically-minded interpretation of the models, not one based on purely contemporary demographics.

**Supplementary Tables and Figures**

All SI tables and figures are available at https://osf.io/3k6dt. Below are brief descriptions of each table and figure, as well as direct links to each.

**Table S1:** *Predicting County-Level Firearms Ownership from Historical Patterns of Slavery (Models Restricted to Counties with Greater Than 25,000 People)*.

This table replicates Table 1 in the main text, restricting the sample to just those counties with greater than 25,000 residents (using the gun-ownership identification criterion of ref. 50).

The table can be found at https://osf.io/qybxp

**Table S2:** *Predicting County-Level Firearms Ownership from Historical Patterns of Slavery (Models Using a White-Only Proxy Variable)*

This table replicates Table 1 in the main text, and Table S1 above, restricting firearms-ownership proxy to just suicides-by-firearm committed by a White individual.

The table can be found at https://osf.io/hgwt6

**Table S3:** *Mediation Output*

This table contains the mediation output (*ab* path, *c’* path, and *c* path) for models in the main text (mediating the direct relationship between either slavery or ruggedness and our gun-ownership proxy), plus additional specifications that include restricting the underlying sample of counties to those with greater than 25,000 residents, specifications that use all counties in the sample, and specifications that use all counties and no controls. In addition, this table provides the mediation output for two alternate mediators - the present-day frequency of people in the county feeling much anger in the previous day (“Anger”), and the present-day frequency of people in the county agreeing that they are able to use their strengths every day (“Self Respect”). Both alternate mediators come from the Gallup Daily Tracking Poll.

The table can be found at https://osf.io/zv6ry

**Table S4:** *Mediation Output (Models Using a White-Only Proxy Variable)*

This table replicates Table S3 above, restricting firearms-ownership proxy to just suicides-by-firearm committed by a White individual.

The table can be found at https://osf.io/7y3m9

**Table S5:** *Predicting a Present-Day Sense of Safety*

This table shows the regression output for the relationship between slavery, geographic ruggedness and present-day feelings of safety (the *a* pathway of the mediations presented in-text). Models include the one presented in the main text, as well as additional specifications that include restricting the underlying sample of counties to those with greater than 25,000 residents, specifications that use all counties in the sample, and specifications that use all counties and no controls.

The table can be found at https://osf.io/ykn2r

**Table S6:** *Present Day Sense of Safety Predicting County-Level Firearms Ownership*

This table shows the regression output for the relationship between present-day feelings of safety and present-day firearms ownership (the *b* pathway of the mediations presented in-text). Models include the one presented in the main text, as well as additional specifications that include restricting the underlying sample of counties to those with greater than 25,000 residents, specifications that use all counties in the sample, and specifications that use all counties and no controls. In addition, this table shows the same output when the firearms-ownership proxy is restricted to just suicides-by-firearm committed by a White individual.

The table can be found at https://osf.io/wr6t3

**Table S7:** *The Moderating Effect of Southern-County-Status on the Relationship Between Feelings of Safety and the Gun Ownership Proxy.*

This table provides the full regression output for the moderation of the relationship between present-day feelings of safety and the present-day gun-ownership proxy by Southern-county-status (presented in Figure 2). In addition, the table presents alternate specifications of the relationship, including models using the White-only gun-ownership proxy, models using all counties, models without controls, and models restricted to counties greater than 25,000 people.

The table can be found at https://osf.io/tm4ay

For the accompanying **Figure S1**, plotting the shape of the interaction for each model of Table S7 (recreating the form of Figure 2), see https://osf.io/h8kmc

**Table S8**: *Predicting County-Level Firearms Ownership from Social-Connectedness Indices (Models Using a White-Only Proxy Variable)*

This table recreates Table 2 from the main text, restricting the gun-ownership proxy to just suicides-by-firearm committed by a White individual.

The table can be found at https://osf.io/54btd

**Table S9:** *Predicting County-Level Firearms Ownership from Social-Connectedness Indices (Models Restricted to Counties with Greater Than 25,000 People)*

This table recreates Table 2 from the main text, using specifications that restrict the sample to counties with more than 25,000 residents.

The table can be found at https://osf.io/at8sb

**Table S10:** *The Moderating Effect of the Slavery Social-Connectedness Index on the Relationship Between Feelings of Safety and the Gun Ownership Proxy.*

This table provides the full regression output for the moderation of the relationship between present-day feelings of safety and the present-day gun-ownership proxy by the Slavery Social-Connectedness Index (presented in Figure 3). In addition, the table presents alternate specifications of the relationship, including models using the White-only gun-ownership proxy, models using all counties, models without controls, and models restricted to counties greater than 25,000 people.

The table can be found at https://osf.io/mkyga

For the accompanying **Figure S2**, plotting the shape of the interaction for each model of Table S10 (recreating the form of Figure 3), see https://osf.io/s6h8u

**Table S11**: *Descriptive Statistics*

This table provides descriptive statistics, and histograms, for all variables.

The table can be found at https://osf.io/7gjxb

**Figure S3:** *Correlations*

This figure presents a correlation matrix between all variables.

The figure can be found at https://osf.io/pgrby

**Table S12:** *Sources for All Variables*

This table contains the citations and wording of all variables used in the manuscript. The table can be found at https://osf.io/etqcs
